# Effects of Subthalamic Nucleus Stimulation on Emotional Prosody Comprehension in Parkinson's Disease

**DOI:** 10.1371/journal.pone.0019140

**Published:** 2011-04-28

**Authors:** Carolin Brück, Dirk Wildgruber, Benjamin Kreifelts, Rejko Krüger, Tobias Wächter

**Affiliations:** 1 Department of Psychiatry and Psychotherapy, University of Tübingen, Tübingen, Germany; 2 Center of Neurology and Hertie Institute for Clinical Brain Research, University of Tübingen, Tübingen, Germany; 3 German Center for Neurodegenerative Diseases (DZNE), Tübingen, Germany; The University of Chicago, United States of America

## Abstract

**Background:**

Although impaired decoding of emotional prosody has frequently been associated with Parkinson's disease (PD), to date only few reports have sought to explore the effect of Parkinson's treatment on disturbances of prosody decoding. In particular, little is known about how surgical treatment approaches such as high frequency deep brain stimulation (DBS) affect emotional speech perception in patients with PD. Accordingly, the objective of this study was to evaluate the effect of subthalamic nucleus (STN) stimulation on prosody processing.

**Methodology/Principal Findings:**

To this end the performance of 13 PD patients on three tasks requiring the decoding of emotional speech was assessed and subsequently compared to the performance of healthy control individuals. To delineate the effect of STN-DBS, all patients were tested with stimulators turned on as well as with stimulators turned off. Results revealed that irrespective of whether assessments were made “on” or “off” stimulation, patients' performance was less accurate as compared to healthy control participants on all tasks employed in this study. However, while accuracy appeared to be unaffected by stimulator status, a facilitation of reactions specific to highly conflicting emotional stimulus material (i.e. stimulus material presenting contradicting emotional messages on a verbal and non-verbal prosodic level) was observed during “on” stimulation assessments.

**Conclusion:**

In sum, presented results suggest that the processing of emotional speech is indeed modulated by STN-DBS. Observed alterations might, on the one hand, reflect a more efficient processing of highly conflicting stimulus material following DBS. However, on the other hand, given the lack of an improvement in accuracy, increased impulsivity associated with STN stimulation needs to be taken into consideration.

## Introduction

Despite the predominance of movement disabilities, symptoms of Parkinson's disease (PD) extend well beyond motor abnormalities [1], [2] including a variety of symptoms pertaining to disturbances in emotion perceptions such as difficulties to infer other peoples' emotional states from facial expressions or body gestures [3], [4].

Aside gestures and facial signals, human beings rely on speech to convey emotions. Spoken words allow access to our thoughts and inner feelings. However, in spoken language the expression of emotional states does not solely depend on the words we use. Often how something is said seems to be more important than what is actually said [5], [6]. Particularly modulations of speech melody (i.e. emotional or affective prosody) convey crucial information about the speaker's current emotional state and his or her true communicative intentions [7].

Several studies point to an impaired perception of emotional prosody in PD, linking the observed deficits to the characteristic degeneration of the basal ganglia associated with PD [8], [9], [10], [11], [12]. Little, however, is known about the effect of PD treatment on the processing of emotional speech.

Aside pharmacological interventions, neurosurgical treatment options such as deep brain stimulation (DBS) of the subthalamic nucleus (STN) assume increasing significance in the management of PD. Particularly for patients in advanced stages of the disease, STN-DBS has become a common treatment [13] associated with marked improvements in motor abilities [14], [15], [16], [17], [18], [19], [20] and quality of life [21], [22], [23]. Although the therapeutic benefits and side effects of STN-DBS have been studied extensively, only relatively few reports have sought to investigate how STN stimulation affects emotion perception.

One of the first reports concerning emotion perception following STN-DBS was published by Dujardin and collaborators [24]. Using an emotional facial expression decoding task, the performance of PD patients was assessed one month before and three months after STN-DBS surgery. While patients showed no impairment of facial affect decoding before surgery, their ability to identify emotional facial expressions was significantly reduced following STN-DBS treatment. Since the observed deficits could not be attributed to secondary variables such as cognitive or visuospatial dysfunctions, depression or anxiety, the authors concluded that STN-DBS induces deficits in the recognition of emotional facial expressions. Several studies have since documented similar impairments following STN-DBS [25], [26], [27], [28].

However, research regarding the effect of STN-DBS on emotion decoding has largely been limited to the perception of emotional facial expressions. Only recently Péron and co-workers [29] have broadened the scope by addressing whether observed impairments translate to the domain of emotional prosody decoding as well. Péron and coworkers assessed the ability to decode vocally expressed emotions in a group of 21 PD patients treated with STN-DBS and compared their performance to 21 PD patients in a pre-operative state and 21 healthy control participants. Results suggest that STN-DBS affects not only the recognition of emotional facial expression, but seems to also modify the decoding of emotions conveyed by the human voice. On closer examination DBS patients' performance appeared to display a systematic emotional bias reflected in a tendency to perceive particularly negative emotional information more strongly [29].

One important limitation of Péron et al's study, however, lies in the use of comparisons between pre- and post-surgical patients to draw conclusions about the effect of STN-DBS. Surgery itself could have a distinct impact on neuropsychological functioning and hence surgery alone could provoke alterations in the decoding of emotional prosody. Only recently a study conducted by Okun and co-workers [30] has drawn attention to the role of surgical procedure in explaining performances changes observed in STN-DBS patients. By comparing patients' performance seven months past DBS implantation to a baseline established for each patient prior to surgery, Okun and collaborators demonstrated marked impairments in verbal fluency following surgery. More importantly, observed deficits remained constant regardless of stimulator status and persisted even during “off” stimulation testing, suggesting not STN-DBS itself but rather insertion or lesion effects associated with electrode implantation as possible underlying mechanisms of impairment [30].

Accordingly, we opted to assess the effect of STN-DBS on emotional prosody independent of effects of surgical procedure. To this end, a group of PD patients treated with STN-DBS was asked to perform a prosody identification task once with STN stimulation turned on as well as once with stimulation turned off. PD patients' performance was subsequently compared to a group of healthy control participants matched for age and formal education. Furthermore, in order to evaluate whether changes in decoding are confined to the appraisal of prosodic elements of speech or whether they apply to the evaluation of emotional information or spoken language in a broader sense, two additional tasks, vowel identification and semantics (i.e. word content) identification, were administered. On the basis of the literature reviewed above, we hypothesized, that PD patients (independent of stimulator status) would show impairments in the decoding of emotional prosody and we expected that patients' abilities to decode prosodic cues would be altered by STN-DBS.

## Materials and Methods

### Participants

13 patients (3 female, 10 male) treated with STN-DBS for PD participated in this study. All patients were recruited from the Department of Neurology of the University of Tübingen. Mean disease duration was 15 years (standard deviation: 6 years). Mean time since STN-DBS implantation was 30 months (standard deviation: 27 months). In three patients PD initially presented as dominant on the right and in ten patients as dominant on the left side. All patients were assessed after overnight withdrawal of antiparkinsonian medication. As all patients were treated with levodopa with a half-life of approximately 50 minutes and only 6 patients additionally received dopamine agonists with a half-life of approximately 6 hours, overnight withdrawal was implemented in order to reduce confounding effects of antiparkinsonsian medication. Severity of motor symptoms was evaluated using the Unified Parkinson's Disease Rating Scale (UPDRS) Part III [31] with assessments made for each patient once with STN stimulation turned on (STN-DBS-ON) and once with STN stimulation turned off (STN-DBS-OFF). Patients' scores on the UPDRS Part III averaged to 23.15 (±8.91) with STN-DBS-ON, whereas patients' scores with STN-DBS-OFF averaged to 56.77 (±11.20) indicating a clinical relevant motor improvement induced by STN-DBS. Patients' performance was compared to a group of 11 healthy controls (HC, 3 female, 8 male) matched for age and years of formal education on a group level.

All participants were native speakers of German and reported no history of psychiatric or neurological illness (with exception of PD in the patient group). In order to exclude individuals with dementia, depression or hearing impairments, all volunteers were screened for cognitive impairments (Mini Mental State Examination [32], inclusion criterion: MMSE>25), depressive symptoms (Becks Depression Inventory [33], inclusion citerion BDI < 11), and hearing loss (pure tone audiometric screening). Demographic and neuropsychological data for both groups are presented in Table 1.

**Table 1:** Demographic and neuropsychological data (mean ± standard deviation) for the Parkinson's disease patients group and the group of healthy control participants.

	PD PATIENT GROUP	HEALTHY CONTROL GROUP
	(N = 13)	(N = 11)
AGE [YEARS]	60.46±8.88	60.09±5.17
YEARS OF FORMAL EDUCATION	13.73±2.67	14.41±3.43
MINI MENTAL STATE EXAMINATION	29.46±0.97	29.55±0.59
BECK DEPRESSION INVENTORY	3.08±2.36	3.00±3.19

### Ethics Statement

The study was conducted according to the Declaration of Helsinki and approval was granted by the ethics committee of the University of Tübingen. Written informed consent was obtained from each participant prior to enrolment in this study.

### Stimulus Material and Tasks

Participants were asked to complete three different tasks: a) vowel identification during which subjects were asked to indicate, whether a spoken word contained the letter “a” or the letter “o” or whether neither “a” nor “o” were present; b) prosody identification during which the participants were asked to judge the emotion conveyed by a speaker's tone of voice using a forced-choice selection of three categories happy, neutral or angry; c) semantics identification during which subjects had to classify each stimulus according to its emotional word content using one of three categories: positive, neutral or negative word meaning.

The same set of stimuli was used for each task. Stimulus material consisted of 60 single German words (30 adjectives, 30 nouns) spoken by 6 actors (3 male, 3 female) either in a happy, an angry or a neutral tone of voice. Adjectives and nouns were selected from a large pool of words previously rated by 45 healthy native- speakers of German according to word valence, arousal and concreteness [34]. Based on the reported ratings of word valence made on a nine-point self-assessment manikin scale [35] ranging from 1 =  highly positive to 9 =  highly negative, 20 words with positive (mean valence rating < 4), 20 with negative (mean valence rating > 6) and 20 with neutral word meaning (mean valence rating between 4 and 6) were selected. Of those words selected, 20 contained the letter “a”, 20 the letter “o” and 20 neither the letter “a” nor the letter “o”. Furthermore 20 words were spoken in a happy, 20 in a neutral and 20 in an angry tone of voice. To ensure that prosody was manipulated as intended, stimulus material was pretested in two pilot studies (pilot study 1: 20 subjects, 10 male, 10 female; pilot study 2: 10 subjects, 5 male, 5 female) and stimuli were only considered for use in the main experiment if the prosodic category was correctly identified by at least 70% of the subjects participating in the pilot studies. Of those 60 words selected 23 words conveyed the same emotional meaning both in word content and prosody (congruent trials). For the remaining 37 words emotional meaning conveyed by prosody and word content did not match (incongruent trials). All stimuli were digitally recorded and normalized to the same mean intensity. Stimulus duration averaged to 0.87 s with a standard deviation of SD = 0.23 s and a range of 0.48 s to 1.44 s.

### Procedure

Since participants had received a detailed written description of purpose and procedure of the study prior to their enrolment, testing always started with a verbal explanation of the study's course of action. All patients were asked to perform three tasks (see above description) once with their stimulators turned on (STN-DBS-ON) and once with their stimulators turned off (STN-DBS-OFF). Changes in stimulator status were made 45 minutes prior to the assessment of task performance. Six of the patients started testing off- stimulation, seven started with stimulators turned on. In order to keep the procedure similar, the performance of control participants was evaluated twice for each task with an interval of about 45 minutes in between repetitions. Task order was balanced between individuals and repetitions.

Participants were tested individually in a quiet laboratory environment. Task requirements, reaction categories and handling of answer equipment were explained immediately before starting each task.

Stimulus delivery and data collection was accomplished using the experimental control software Presentation (Neurobehavioral Systems Inc., Albany, CA, USA) installed on a portable computer equipped with high-quality headphones (Sennheiser HD515, Sennheiser, Germany). Volume of stimulus presentation was adjusted to comfortable hearing levels by the participants themselves. Stimuli within each task were fully randomized. Participants indicated their answers by pressing a button on a Cedrus RB-730 response pad (Cedrus Corporation, San Pedro, CA, USA). Buttons were clearly labelled and visual aid for each response category was provided on the computer screen. No time limitations for answers were predetermined. Moreover, by pressing a repeat button participants had the opportunity to have a stimulus replayed in case of indecision. Necessary intermissions in the course of action were used to gather demographical, medical and neuropsychological background data of the participants.

### Data Analysis

Subjects' performance was evaluated using mean reactions times and accuracy rates ( =  number of stimuli correctly identified divided by the number of stimuli presented) as measures of general performance.

In order to determine the effect of STN-DBS, performance measures of STN-DBS patients were subjected to a 3×2×3×3 repeated- measures analysis of variance (ANOVA) with task (vowel identification/prosody identification/semantics identification), stimulator status (STN-DBS-ON/STN-DBS-OFF), prosodic category (happy/angry/neutral) and word content category (positive/negative/neutral) as within- subject- factors.

Differences between the patient group and the group of healthy control participants were **assessed** using a 2×3×3×3 repeated- measures ANOVA with group (PD/HC) as between-subject-factor and task (vowel identification/prosody identification/semantics identification), prosodic category (happy/angry/neutral) and word content category (positive/negative/neutral) as within-subject-factors.

To account for violations of sphericity, p-values were Greenhouse-Geisser corrected. Significant main effects were further examined using paired samples t-test. In cases where the employed ANOVA revealed a significant interaction of prosodic category and word content, post-hoc comparisons between congruent and incongruent trials were conducted. Trials during which both sources of emotional information (i.e. prosody and word content) carried the same emotional meaning (e.g. word with positive content spoken happily) were regarded as congruent. Trials during which both prosody and word content were not coherently linked (e.g. word with positive content spoken angrily) were considered incongruent. To probe the data in greater detail incongruent trials were further divided into high conflict trials or low conflict trials based on the degree of discrepancy between presented verbal and prosodic information. Incongruent trials were regarded as *high conflict* if emotional prosody and word content proved to be contradictory (i.e. word with positive word content spoken in an angry tone of voice or word with negative word content spoken in a happy tone of voice). Incongruent trials were regarded as *low conflict* if neutral word meaning was paired with happy or angry prosody or vice versa if neutral prosody was paired with positive or negative word meaning. To further investigate interactions between task, prosodic category and word content, differences in performance measures between congruent and high conflict trails as well as between congruent and low conflict trials, and low conflict and high conflict trials were computed for each task and subsequently compared among tasks using paired samples t-tests.

## Results

Mean accuracy rates and reaction times obtained for the PD group as well as the HC group are displayed in Table 2.

**Table 2:** Accuracy rates (mean ± standard deviation) and reactions times (mean ± standard deviation; measurement unit: ms) obtained for the PD patients group and the group of healthy control participants within each task (PI =  prosody identification, SI =  semantics identification, VI  =  vowel identification).

TASK	PD PATIENT GROUP				HEALTHY CONTROL GROUP	
	ACCURACY RATE		REACTION TIME		ACCURACY RATE	REACTION TIME
	STN-DBS-ON	STN-DBS-OFF	STN-DBS-ON	STN-DBS-OFF		
**PI**	0.71±0.23	0.72±0.20	2250±578	2649±779	0.87±0.09	2336±646
**SI**	0.78±0.15	0.81±0.19	2555±824	2792±697	0.91±0.04	2518±845
**VI**	0.95±0.05	0.95±0.06	2259±614	2561±474	0.97±0.02	2012±418

### Comparisons between “On” and “Off” Stimulation Assessments

#### Analysis of accuracy rates

The analysis of accuracy rates failed to reveal a significant main effect of STN-DBS [F (1.00, 12.00) = 1.57, p = 0.234]. Mean accuracy rates, thus, did not differ significantly between STN-DBS-ON and STN-DBS-OFF assessments. However, significant main effects of task [F (1.99, 23.90) = 10.66, p = 0.000] and prosodic category [F (1.86, 22.33) = 3.56, p = 0.049] were observed. Results of post- hoc comparisons computed to further delineate the respective main effects are summarized in Table 3. Post-hoc inspections of the main effect of task revealed that regardless of stimulator status both the prosody identification task (M = 0.72, SD = 0.20) as well as the semantics identification task (M = 0.80, SD = 0.17) were associated with more errors as compared to vowel identification (M = 0.95, SD = 0.05). Moreover, post- hoc analysis of the main effect of prosodic category indicated that irrespective of task condition stimuli spoken in a neutral tone of voice (M = 0.86, SD = 0.10) were classified with a higher accuracy relative to stimuli spoken in a happy tone of voice (M = 0.80, SD = 0.14).

**Table 3:** Results of post-hoc comparisons computed for significant main effects and interactions obtained from the STIMULATOR STATUS × TASK × PROSODIC CATEGORY × WORD CONTENT ANOVA with accuracy rates as dependent variable.

POST-HOC ANALYSIS FOR	COMPARISON	P-VALUE
**MAIN EFFECT OF TASK**		
	PI–SI	n.s.
	PI–VI	p<0.01
	SI–VI	p<0.05
**MAIN EFFECT OF PROSODIC CATEGORY**		
	happy–neutral	p<0.05
	happy–angry	n.s.
	neutral–angry	n.s
**PROSODIC CATEGORY × WORD CONTENT**		
	CT–HT	p<0.01
	CT–LT	p<0.05
	HT–LT	p<0.01
**TASK × PROSODIC CATEGORY × WORD CONTENT**		
CT-HT-DIFFERENCES	PI–SI	n.s.
	PI–VI	p<0.01
	SI–VI	n.s.
CT-LT-DIFFERENCES	PI–SI	n.s.
	PI–VI	p<0.05
	SI–VI	n.s.
LT-HT-DIFFERENCES	PI–SI	n.s.
	PI–VI	n.s.
	SI–VI	n.s.

PI =  prosody identification, SI =  semantics identification, VI =  vowel identification; CT =  congruent trials,HT =  high conflict trials, LT =  low conflict trials, n.s. =  not significant

No significant interactions involving stimulator status were revealed. However, data analysis did indicate a significant interaction of prosodic category and word content [F(1.80, 21.64) = 11.98, p = 0.000] as well as a trend-level interaction of task, prosodic category and word content [F(3.40, 40.78) = 2.71, p = 0.051]. To further delineate interaction effects including prosodic category and word content pair-wise comparisons among congruent, high and low conflict trials were conducted using paired samples t-tests. Results of computed t-test comparisons are summarized in Table 3. Mean accuracy rates corresponding to congruent, low conflict and high conflict trials as well as mean accuracy rate differences between the different trial types are displayed in Table 4. Results revealed higher accuracy in judging congruent trials as compared to high conflict or low conflict trials. Moreover, significantly higher accuracy rates were observed for low conflict as compared to high conflict trials. As indicated by the task × prosodic category × word content interaction, congruency effects appeared to be task-specific. To further delineate task-specificity, differences in accuracy rates between congruent trials and high conflict trials as well as differences between congruent trials and low conflict trials, and low conflict and high conflict trials were computed for each task and subsequently compared among tasks using paired samples t-tests. Results indicated higher accuracy in judging congruent trials as compared to high conflict trials for all three tasks with significantly larger mean accuracy differences between congruent and high conflict trials obtained for prosody identification as compared to vowel identification. Moreover, during prosody identification low conflict trials were associated with more errors as compared to congruent trials, whereas during vowel identification both congruent and low conflict trials were classified with similar precision leading to significantly smaller difference values between congruent and low conflict trials obtained for vowel identification as compared to prosody identification. No significant effects were observed for comparisons computed using differences values between low conflict and high conflict trials.

**Table 4:** Accuracy rates (mean ± standard deviation) corresponding to congruent (CT), low conflict (LT) and high conflict trials (HT) as well as accuracy rate differences (mean ± standard deviation) between the different trial types obtained within each task (PI =  prosody identification, SI =  semantics identification, VI =  vowel identification).

	PD PATIENT GROUP			
	PI	SI	VI	OVERALL
**CT**	0.83±0.14	0.87±0.12	0.95±0.06	0.88±0.08
**HT**	0.58±0.28	0.70±0.26	0.91±0.09	0.73±0.17
**LT**	0.71±0.24	0.79±0.21	0.97±0.04	0.82±0.13
**CT-HT**	0.25±0.19	0.17±0.21	0.04±0.08	
**CT-LT**	0.12±0.18	0.08±0.19	−0.02±0.04	
**LT-HT**	0.13±0.11	0.09±0.10	0.06±0.06	

Note: Slight discrepancies between (CT-HT)/(CT-LT)/(LT-HT) subtraction values and mean difference values reported in this table are due to rounding.

#### Analysis of reactions times

No significant main effect of stimulator status was observed [F(1.00, 12.00) = 3.55, p = 0.084]. Mean reaction times, thus, did not differ significantly between STN-DBS-ON and STN-DBS-OFF assessments. The analysis of reaction times, however, yielded a significant main effect of prosodic category [F(1.73, 20.73) = 8.61, p = 0.003] and word content [F(1.43, 17.21) = 4.11, p = 0.046]. Results of post- hoc comparisons computed for each significant main effect are summarized in Table 5. Post-hoc analysis of the main effect of word content indicated slower reactions following stimulus material with negative word content (M = 2580 ms, SD = 459 ms) as compared to stimuli with positive word content (M = 2452 ms, SD =  453 ms). The main effect of emotional prosody was explained by slower reactions to angrily (M = 2625 ms, SD = 445 ms) as compared to happily (M = 2467 ms, SD = 474 ms) or neutrally (M = 2444 ms, SD = 444 ms) spoken words.

**Table 5:** Results of post-hoc comparisons computed for significant main effects and interactions obtained from the STIMULATOR STATUS × TASK × PROSODIC CATEGORY × WORD CONTENT ANOVA with reaction times as dependent variable.

POST-HOC ANALYSIS FOR	COMPARISON	P-VALUE
**MAIN EFFECT OF PROSODIC CATEGORY**		
	happy–neutral	n.s.
	happy–angry	p<0.05
	neutral–angry	p<0.01
**MAIN EFFECT OF WORD CONTENT**		
	pos –neg	p<0.01
	pos – neu	n.s.
	neg– neu	n.s.
**TASK × WORD CONTENT**		
MAIN EFFECT OF WORD CONTENT FOR VI	VI: pos–neg	n.s.
	VI: pos–neu	p<0.01
	VI: neg–neu	p<0.05
**PROSODIC CATEGORY × WORD CONTENT**		
	CT–HT	p<0.01
	CT–LT	n.s.
	HT–LT	p<0.05
**TASK × PROSODIC CATEGORY × WORD CONTENT**		
CT-HT-DIFFERENCES	PI–SI	n.s.
	PI–VI	n.s.
	SI–VI	n.s.
CT-LT-DIFFERENCES	PI–SI	n.s.
	PI–VI	p<0.05
	SI–VI	p<0.01
LT-HT-DIFFERENCES	PI–SI	n.s.
	PI–VI	n.s.
	SI–VI	n.s.
**STIMULATOR × PROSODIC CATEGORY × WORD CONTENT**		
	CT: ON–OFF	n.s.
	HT: ON–OFF	p = 0.05
	LT: ON–OFF	n.s.
	(LT-HT): ON-OFF	n.s.

PI =  prosody identification, SI =  semantics identification, VI =  vowel identification; CT =  congruent trials, HT =  high conflict trials, LT =  low conflict trials, pos =  positive, neg =  negative, neu  =  neutral, n.s. =  not significant

A significant interaction between stimulator status, prosodic category and word content was observed [F(3.54, 42.46) = 3.06, p = 0.031]. To further investigate this interaction, comparisons between reaction times obtained during STN-DBS-ON and STN-DBS-OFF were computed separately for congruent, low conflict, and high conflict trials using paired-samples t-tests. Corresponding results are summarized in Table 5. Results indicated a significant ON-OFF- difference for high conflict trials, explained by faster reactions observed during STN-DBS-ON (M = 2456 ms, SD = 517 ms) as compared to STN-DBS-OFF (M = 2889 ms, SD = 700 ms). ON vs. OFF comparisons computed for congruent and low conflict trials, failed to reach statistical significance. Moreover, reaction time differences between low conflict and high conflict trials were computed and subsequently compared between STN-DBS-ON and STN-DBS-OFF using paired samples t-tests. Results revealed a trend-level difference (t(12) =  1.94, p =  0.08) with a mean difference value of −90 ms (SD = 239 ms) obtained for STN-DBS-ON and a mean difference value of −260 ms (SD = 308 ms) for STN-DBS-OFF. Additionally, the results indicated a significant interaction of task × word content [F(2.22, 26.64) = 3.63, p = 0.036], prosodic category × word content [F(2.73, 32.86) = 8.48, p = 0.000] and task × prosodic category × word content [F(3.14, 37.72) = 3.08, p = 0.037]. For post-hoc investigation of the task × word content interaction, each task was analyzed separately using a repeated-measures ANOVA with word content as within-subject variable. Paired samples t-test helped to further delineate the findings. Corresponding results are summarized in Table 5. A significant main effect of word content was obtained only for vowel identification [F(1.69, 20.35) = 6.02, p = 0.012], which was explained post-hoc by faster responses following stimulus material with neutral (M = 2337 ms, SD = 470 ms) as compared to positive (M = 2457 ms, SD = 439 ms) or negative (M = 2436 ms, SD = 500 m) word content. Results obtained for semantics identification indicated a trend-level main effect of word content [F(1.30, 15.56) = 4.09, p = 0.051]. Statistics computed for prosody identification failed to reach significance. Post-hoc inspections of interactions between prosodic category and word content were done using comparisons between congruent, high conflict and low conflict trials. Results of computed comparisons are summarized in Table 5. Mean reaction times corresponding to congruent, low conflict and high conflict trials as well as mean reaction times differences between the different trial types are displayed in Table 6. Results again indicated an advantage of congruency with faster reactions to congruent as compared to high conflict trials. Moreover, a significant reaction time difference between high conflict and low conflict trials was revealed, with slower reactions obtained for high conflict as compared to low conflict trials. To further investigate the task × prosodic category × word content interaction, differences between congruent and high conflict trials, as well as between congruent and low conflict trials, and low conflict and high conflict trials were computed for each task and subsequently compared among tasks using paired samples t-tests. Closer inspection of significant effects revealed slower reactions following low conflict trials as compared to congruent trials for both prosody identification and semantics identification, whereas during vowel identification slower reactions following congruent trials as compared to low conflict trials were observed.

**Table 6:** Reaction times (mean ± standard deviation, measurement unit: ms) corresponding to congruent (CT), low conflict (LT) and high conflict trials (HT) as well as reaction time differences (mean ± standard deviation, measurement unit: ms) among trials obtained within each task (PI =  prosody identification, SI =  semantics identification, VI =  vowel identification).

	PD PATIENT GROUP			
	PI	SI	VI	OVERALL
**CT**	2335±555	2509±655	2419±500	2421± 417
**HT**	2624±636	2812±810	2581±442	2672± 495
**LT**	2448±587	2728±700	2317±462	2497± 463
**CT-HT**	−288±228	−303±277	−162±153	
**CT-LT**	−112±194	−218±252	103±148	
**LT-HT**	−175±241	−85±443	−265±130	

Note: Slight discrepancies between (CT-HT)/(CT-LT)/(LT-HT) subtraction values and mean difference values reported in this table are due to rounding.

### Comparisons between the PD Group and Healthy Control Group

#### Analysis of Accuracy Rates

Analysis of accuracy rates yielded a significant main effect of group [F(1, 22) = 6.71, p = 0.017] revealing PD patients' performance overall to be less accurate as compared to healthy control participants. Patients' accuracy rates across all three tasks averaged to 0.82 (SD = 0.24), whereas healthy control individuals achieved an accuracy rate of 0.92 (SD =  0.12) on average. Consistent with results of the intragroup analysis reported above, a significant main effect of task [F (1.95, 42.97) = 16.55, p = 0.000] was observed. Results of post- hoc comparisons computed to further delineate the main effect are summarized in Table 7. Post- hoc analysis indicated higher accuracy during vowel identification (M = 0.96, SD = 0.04) as compared to prosody identification (M = 0.79, SD = 0.18) and semantics identification (M = 0.85, SD = 0.14).

**Table 7:** Results of post-hoc comparisons computed for significant main effects and interactions obtained from the GROUP × TASK × PROSODIC CATEGORY × WORD CONTENT ANOVA with accuracy rates as dependent variable.

POST-HOC ANALYSIS FOR	COMPARISON	P-VALUE
**MAIN EFFECT OF **TASK		
	PI–SI	n.s.
	PI–VI	p<0.01
	SI–VI	p<0.01
**PROSODIC CATEGORY × WORD CONTENT**		
	CT–HT	p<0.01
	CT–LT	p<0.05
	HT–LT	p<0.01
**GROUP × PROSODIC CATEGORY × WORD CONTENT**		
	(CT–HT): PD-HC	p<0.05
	(CT–LT): PD-HC	n.s.
	(HT–LT): PD-HC	p<0.05
**TASK × PROSODIC CATEGORY × WORD CONTENT**		
CT-HT-DIFFERENCES	PI–SI	p<0.05
	PI–VI	p<0.01
	SI–VI	n.s.
CT-LT-DIFFERENCES	PI–SI	n.s.
	PI–VI	p<0.01
	SI–VI	p<0.05
LT-HT-DIFFERENCES	PI–SI	n.s.
	PI–VI	n.s.
	SI–VI	n.s.

PI =  prosody identification, SI =  semantics identification, VI =  vowel identification; CT =  congruent trials,

HT =  high conflict trials, LT =  low conflict trials, n.s. =  not significant

A significant interaction between group, prosodic category and word content category was observed [F(2.08, 45.75) = 3.80, p = 0.028]. To further investigate the group × prosodic category × word content category interaction, differences between congruent and high conflict trials, congruent and low conflict trials as well as between low conflict and high conflict trials were calculated and subsequently compared between groups using independent samples t-tests. Results are summarized in Table 7. For both groups mean accuracy rates corresponding to congruent, low conflict and high conflict trials as well as mean accuracy rate differences among each trial type are displayed in Table 8. Comparisons revealed that both groups judged congruent trials with higher accuracy as compared to high conflict and low conflict trials. However, larger mean accuracy rate differences between high conflict and congruent trials as well as between low conflict and congruent trials were observed for PD patients as compared to healthy controls. Furthermore, the results yielded a significant interaction of prosodic category × word content [F(2.08, 45.75) = 14.28, p = 0.000] and task × prosodic category × word content [F(3.98, 87.47) = 4.15, p = 0.004]. All interactions involving prosodic category × word content were further analyzed using comparisons between congruent, low conflict and high conflict trials. Results are summarized in Table 7. Mean accuracy rates corresponding to congruent, low conflict and high conflict trials as well as mean accuracy rate differences between the different trial types are displayed in Table 8. Results again indicated significant differences between congruent and both high conflict and low conflict trials, as well as between low conflict and high conflict trials. Corresponding mean accuracy rates once more pointed to an advantage of congruency as congruent trials were judged with higher precision compared to low conflict and high conflict trials. Comparisons between both types of incongruent trials indicated significantly lower proportions of correct responses for high conflict as relative to low conflict trials. To further investigate the interaction of task × prosodic category × word content, differences between congruent and low conflict trials, as well as between congruent and high conflict trials and low conflict and high conflict trials were calculated and subsequently compared among tasks using paired samples t-test. During both prosody identification and semantics identification low conflict trials were associated with more errors as compared to congruent trials, whereas during vowel identification both congruent and low conflict trials were classified with similar precision as indicated by significantly smaller corresponding mean accuracy rate differences observed for vowel identification as compared to prosody identification and semantics identification. Considering task comparisons computed using differences values between congruent and high conflict trials, results indicated significantly smaller mean difference values for both vowel identification and semantics identification as compared to prosody identification.

**Table 8:** Accuracy rates (mean ± standard deviation) corresponding to congruent (CT), low conflict (LT) and high conflict trials (HT), as well as accuracy rate differences (mean±standard deviation) between the different trial types obtained within each task (PI =  prosody identification, SI =  semantics identification, VI =  vowel identification) and experimental group.

	HEALTHY CONTROLL GROUP				PD PATIENT GROUP				OVERALL			
	PI	SI	VI	OVERALL	PI	SI	VI	OVERALL	PI	SI	VI	OVERALL
**CT**	0.92±0.05	0.92±0.04	0.97±0.03	0.94±0.03	0.83±0.14	0.87±0.12	0.95±0.06	0.88±0.08	0.87±0.12	0.89±0.10	0.96±0.05	0.91±0.07
**HT**	0.83±0.14	0.90±0.09	0.94±0.06	0.89±0.07	0.58±0.28	0.70±0.26	0.91±0.09	0.73±0.18	0.69±0.26	0.79±0.22	0.92±0.08	0.80±0.16
**LT**	0.85±0.13	0.91±0.04	0.99±0.01	0.92±0.05	0.71±0.24	0.79±0.21	0.97±0.04	0.82±0.13	0.77±0.21	0.85±0.16	0.98±0.04	0.87±0.11
**CT-HT**	0.10±0.13	0.02±0.09	0.04±0.06		0.25±0.19	0.17±0.21	0.04±0.08		0.18±0.18	0.10±0.18	0.04±0.07	
**CT-LT**	0.08±0.11	0.00±0.05	−0.02±0.03		0.12±0.18	0.08±0.19	−0.02±0.04		0.10±0.14	0.05±0.14	−0.02±0.03	
**LT-HT**	0.02±0.13	0.01±0.08	0.06±0.06		0.13±0.11	0.09±0.10	0.06±0.06		0.08±0.13	0.05±0.10	0.06±0.05	

Note: Slight discrepancies between (CT-HT)/(CT-LT)/(LT-HT) subtraction values and mean difference values reported in this table are due to rounding. Similarly discrepancies between overall means and corresponding averages of values reported for the patient group and healthy control group are introduced by rounding effects.

#### Analysis of reactions times

Analysis of reaction times revealed no significant main effects or interactions involving group. Patients and controls did not differ with respect to the promptness of their reactions.

However, results yielded a significant main effect of task [F(1.38, 30.40) = 5.21, p = 0.020], indicating significantly slower reactions during semantics identification (M =  2603 ms, SD = 750 ms) as compared to vowel identification (M = 2227 ms, SD = 479 ms) and prosody identification (M =  2397 ms, SD = 597 ms, Table 9). Moreover, similar to the results described for the analysis of STN-DBS effects, a significant main effect of prosodic category [F(1.56, 34.31) = 12.60, p =  0.000] was observed. Post-hoc inspection (Table 9) pointed to slower reactions following stimulus material spoken in an angry (M = 2543 ms, SD = 565 ms) as compared to a happy (M = 2395 ms, SD = 598 ms) or neutral (M = 2290 ms, SD = 450 ms) tone of voice.

**Table 9:** Results of post-hoc comparisons computed for significant main effects and interactions obtained from the GROUP × TASK × PROSODIC CATEGORY × WORD CONTENT ANOVA with reaction times as dependent variable.

POST-HOC ANALYSIS FOR	COMPARISON	P-VALUE
**MAIN EFFECT OF TASK**		
	PI–SI	p<0.05
	PI–VI	n.s.
	SI–VI	p<0.05
**MAIN EFFECT OF PROSODIC CATEGORY**		
	happy – neutral	n.s.
	happy – angry	p<0.01
	neutral - angry	p<0.01
**TASK × WORD CONTENT**		
MAIN EFFECT OF WORD CONTENT FOR VI	VI: pos–neg	n.s.
	VI: pos–neu	p<0.01
	VI: neg–neu	p<0.01
MAIN EFFECT OF WORD CONTENT FOR PI	PI: pos–neg	p<0.01
	PI: pos–neu	n.s.
	PI: neg–neu	p<0.01
**PROSODIC CATEGORY × WORD CONTENT**		
	CT–HT	p<0.01
	CT–LT	p<0.05
	HT–LT	p<0.01
**TASK × PROSODIC CATEGORY × WORD CONTENT**		
CT-HT-DIFFERENCES	PI–SI	n.s.
	PI–VI	p<0.05
	SI–VI	n.s.
CT-LT-DIFFERENCES	PI–SI	n.s.
	PI–VI	p<0.05
	SI–VI	p<0.01
LT-HT-DIFFERENCES	PI–SI	n.s.
	PI–VI	n.s.
	SI–VI	n.s.

PI =  prosody identification, SI =  semantics identification, VI =  vowel identification; CT =  congruent trials,

HT =  high conflict trials, LT =  low conflict trials, pos =  positive, neg =  negative, neu  =  neutral,

n.s. =  not significant

Moreover, results yielded a significant interaction of task × word content [F(1.92, 42.23) = 4.12, p = 0.025], prosodic category × word content [F(2.91, 63.94) = 19.28, p = 0.000] as well as task × prosodic category × word content [F(3.81, 83.88) = 4.30, p = 0.004]. To further investigate the task by word content interaction, each task was analyzed separately using a repeated- measures ANOVA with word content defined as within-subject variable. Post-hoc paired sample t-tests helped to further delineate the findings. Results are summarized in Table 9. Post- hoc analysis indicated a significant main effect of word content for vowel identification [F(1.60, 36.87) = 12.34, p =  0.000] and prosody identification [F(1.86, 42.93) = 5.08, p = 0.012]. During the vowel identification task, faster reactions following stimulus material with neutral (M = 2148 ms, SD = 482 ms) as compared to positive (M = 2263 ms, SD = 485 ms) or negative (M = 2272 ms, SD = 488 ms) word content were observed. During the prosody identification task, reactions were faster following stimulus material with positive (M = 2340 ms, SD = 522 ms) or neutral (M = 2350 ms, SD = 699 ms) as compared to negative (M = 2502 ms, SD = 624 ms) word content. Finally, to further investigate the interactions involving prosodic category and word content, comparisons between congruent, low conflict and high conflict trials were made. Results of the computed comparisons are summarized in Table 9. Mean reaction times corresponding to congruent, low conflict and high conflict trials as well as mean reaction times differences between the different trial types are displayed in Table 10.

**Table 10:** Reaction times (mean±standard deviation, measurement unit: ms) corresponding to congruent (CT), low conflict (LT) and high conflict trials (HT) as well as reaction time differences (mean±standard deviation, measurement unit: ms) between the different trial types obtained within each task (PI =  prosody identification, SI =  semantics identification, VI =  vowel identification) and experimental group.

	HEALTHY CONTROLL GROUP				PD PATIENT GROUP				OVERALL			
	PI	SI	VI	OVERALL	PI	SI	VI	OVERALL	PI	SI	VI	OVERALL
**CT**	2108±564	2359±835	1997±438	2155±570	2335±555	2509±655	2419±500	2421± 417	2231±559	2441±730	2226±510	2299±500
**HT**	2808±970	2678±965	2290±549	2592±719	2624±636	2812±810	2581±442	2672± 495	2708±793	2751±867	2448±505	2636±595
**LT**	2271±585	2558±913	1884±366	2237±583	2448±587	2728±700	2317±462	2497± 463	2367±581	2650±791	2119±467	2378±526
**CT-HT**	−701±544	−319±588	−294±221		−288±228	−303±277	−162±153		−477±447	−310±436	−222±195	
**CT-LT**	−164±180	−199±326	112±148		−112±194	−218±252	103±148		−136± 186	−209±282	107±145	
**LT-HT**	−537±491	−120±737	−406±303		−175±241	−85±443	−265±130		−341±411	−101±582	−329±232	

Note: Slight discrepancies between (CT-HT)/(CT-LT)/(LT-HT) subtraction values and mean difference values reported in this table are due to rounding. Similarly discrepancies between overall means and corresponding averages of values reported for the patient group and healthy control group are introduced by rounding effects.

Results, again, point to an advantage of congruency with faster reactions obtained to congruent as compared to high conflict or low conflict trials. Moreover, a significant reaction time difference between high conflict and low conflict trials was revealed, with slower reactions obtained for high conflict as compared to low conflict trials. To further investigate the interaction task × prosodic category × word content, reaction time differences between congruent, high conflict and low conflict trials were computed for each task and subsequently compared among tasks. Results revealed that both during prosody and semantics identification participants reacted slower to low conflict as compared to congruent trials, whereas during vowel identification slower reactions following congruent trials as relative to low conflict trials were observed. Moreover, results indicated slower reactions following high conflict trials as compared to congruent trials for both vowel identification and prosody identification, with a greater mean reaction time difference observed for prosody identification as compared to vowel identification. Subsequent analysis using difference values computed between low conflict and high conflict trials did not reveal significant results for any further comparison made.

## Discussion

Over the last years compelling evidence concerning an impairment of prosody comprehension in patients suffering from PD has been gathered in the literature. However, to date little is known about how the treatment of PD, particularly STN stimulation, affects alterations in the perception of emotional speech melody. Accordingly, the purpose of this study was to evaluate the effect of STN-DBS on the decoding of emotional speech. Our results identify PD patients' performance overall to be less accurate as compared to the performance of healthy participants, regardless of whether assessments were made with STN-DBS turned on or off. However, while accuracy appeared to be unaffected by stimulator status, a facilitation of reactions specific to trials presenting highly conflicting stimulus material was observed in measurement sessions during which STN-DBS remained active as compared to those sessions during which STN-DBS was switched off.

### Comparisons between “On” and “Off” Stimulation Assessments: Effects of STN-DBS

Based on studies investigating the effect of STN-DBS on the recognition of facial emotional expression and emotional prosody, we predicted that STN-DBS would alter the decoding of emotional prosody. Indeed, our analysis did indicate a significant influence of STN-DBS, affecting not only speech melody decoding but also vowel and word content judgment. Stimulation effects presented as a facilitation of patients' reactions specific to highly conflicting emotional stimulus material during STN-DBS-ON as compared to STN-DBS-OFF testing. Since observed facilitation remained confined to highly conflicting stimulus material only, improvements of motor functions following STN-DBS can be excluded as explanation of this finding. Instead, one could assume, that observed improvements in speed reflect a more efficient processing of highly conflicting stimulus material. Indeed, although the effect of STN-DBS on cognitive and behavioral functioning still lacks consensus, with beneficial, detrimental as well as no effects on various abilities reported in the literature [36], [37], [38], studies comparing cognitive function during “on” and “off” stimulation assessments suggest an improvement of executive functions associated with STN-DBS-ON [39], [40]. Accordingly, the facilitation of reactions to highly conflicting stimulus material observed during STN-DBS-ON assessments could reflect improvements in aspects of executive functioning such as the patients' abilities to inhibit irrelevant stimulus dimensions when faced with competing response alternative presented by conflicting emotional information at a verbal and nonverbal level. But especially in light of lacking increases in performance accuracy this interpretation becomes limited.

An alternative explanation, however, might be provided from recent findings indicating that STN-DBS may contribute to rash or impulsive decision making [41] and more interestingly that impulsive behavior may present especially pronounced when faced with highly conflicting decision choices [42]. For instance, using a simple decision making task, Frank and co-workers not only showed that STN-DBS fosters impulsive responding, but also evidenced that patients while under STN-DBS responded even faster to highly than to lowly conflicting choices indicating that STN-DBS appears to interfere with the ability to slow down when faced with decision conflict [42]. In keeping with these findings, our results might thus reflect a case of STN-DBS-driven increases of impulsivity specific to decisional conflict imposed by highly conflicting stimulus material.

In the context of increased impulsivity one might expect observed facilitations in responding to be paralleled by deteriorations in decision accuracy. Our results, however, fail to reveal a significant effect of STN stimulation on accuracy of decision making. Neither the precision of speech melody decoding nor the accuracy of emotional word content or vowel recognition revealed to be altered following STN-DBS.

A possible explanation for these negative findings could be that the employed delay of approximately 45 minutes between changes in stimulator status and the assessment of task performance does not suffice to fully reduce or restore the effect of STN-DBS. Although we observed distinct changes in motor abilities within this time frame, the effect of STN-DBS on emotional processing might follow a different, more decelerated time course. Residual stimulation effects during STN-DBS-OFF conditions or alternatively the lack of a fully developed stimulation effect during STN-DBS-ON conditions could have clouded our patients' true task performance during both condition and thus could have complicated the detection of possible discrepancies in task performance.

Finally, faced with only very limited data available on speech melody recognition following STN-DBS, not only the issue whether or not STN-DBS affects prosody decoding in general, but also the nature of such potential changes remains an open question. Recent work by Péron and collaborators [29] suggests that STN-DBS might not alter abilities related to the labeling of emotion but rather might impact on the intensity with which emotions are perceived. Aiming to explore the recognition of emotional prosody following STN-DBS, the authors chose two complementary methods to evaluate patients' performance: a) categorical judgments (i.e. performance in terms of percentages of correct reactions), b) continuous judgments (i.e. ratings of the degree to which different emotions were expressed in a given stimulus). Whereas no differences between patients with or without STN-DBS were observed regarding categorical judgments, the investigation of continuous judgments revealed different patterns of reactions for the different groups of participants included in this study. Results indicate that STN-DBS patients perceived emotions with a higher subjective intensity. Alterations in the perception of certain affective properties of a vocal stimulus such as intensity would not directly translate into higher accuracy in classifying emotional speech and consequently would not be easily detected by labeling task such as the ones employed in this study.

### Comparisons between the PD Patient Group and Healthy Control Group

In accordance with previous research, we hypothesized that PD patients, in general, would show marked deficits in the decoding of emotional prosody. Indeed, analysis of accuracy scores revealed a poorer overall performance for PD patients as compared to healthy controls, thereby indicating an impaired ability to classify the employed stimulus material correctly. At first glance, these findings seem to replicate reports about disturbances of prosody perception associated with PD [8], [9], [10], [11], [12]. However, given the lack of task-specificity of those observed group differences, our data does not suggest a specific impairment of emotional prosody processing, but rather indicates a more general cognitive problem equally relevant to all three tasks employed in this study. As discussed by Breitenstein and colleagues [8] one such general cognitive factor contributing not only to an impairment of prosody perception, but also to deficits in other domains such as, for instance, vowel and word content judgment might be executive dysfunction. Impairments of executive functioning among patients suffering from PD have frequently been reported in the literature [43], [44], [45]. Although the term executive functions is associated with a myriad of cognitive abilities devoted to the planning and organization of behaviour, the most fundamental executive processes appear to be focusing attention to relevant while inhibiting irrelevant information [46].

With respect to the three task employed in this study, successful performance on each task requires participant to focus attention on task-relevant aspects of presented stimuli (e.g. prosodic features during the emotional prosody task) while suppressing other stimulus dimension (e.g. word content during the emotional prosody task), thus PD- associated disturbances in the patients' abilities to focus attention and inhibit interferences could have lead to observed impairments in task performance.

As reasoned by Bowers et al. [47], if patients had difficulties to maintain attention focus and thus were to be distracted by concomitant task- irrelevant stimulus dimensions, then task performance should be affected especially when faced with incongruent as compared to congruent stimulus material. This assumption is generally supported by our data: Asked to judge incongruent stimulus material, participants from both the HC and PD group responded more slowly and less accurately. However, comparisons computed between both groups revealed that PD patients relative to healthy individuals experienced even greater difficulties in judging incongruent trials: Our analysis indicated significantly larger mean accuracy rate differences between congruent and incongruent trials for PD patients when compared to healthy controls.

Finally, although described results appear to fit well within the existing body of research concerning cognitive alterations in Parkinson's, one needs to bear in mind that observed deficits might also reflect lesion effects associated with the surgical implantation of DBS electrodes rather than genuine symptoms of PD. Comparisons to pre-surgical neuropsychological profiles would have helped to determine effects of surgery, however, no such data is available for the patients enrolled in this study.

### Conclusion

In conclusion, aiming to investigate the effect of STN-DBS on prosody decoding, the present study revealed an impact of STN stimulation on the speed of patients' reactions, whereas accuracy proved to be unaffected by stimulator status. Interestingly, the facilitation of reactions remained restricted to highly conflicting stimulus material only, excluding improvements in motor functioning as a simple explanation. Instead, observed alterations might reflect a more efficient processing of highly conflicting stimulus material following DBS. However, given the lack of an improvement in accuracy, increased impulsivity associated with STN stimulation needs to be taken into consideration. Thus in sum, it appears that the processing of emotional speech is indeed modulated by STN-DBS, however, the nature of such modulation require further elaboration. Particularly with respect to several limitations of this study, such as, for instance, the small sample size or the lack of pre-surgical data to control for confounding effects of electrode implantation, further studies a warranted to detail presented findings.
